# HCV Genotype Has No Influence on the Incidence of Diabetes—EpiTer Multicentre Study

**DOI:** 10.3390/jcm11020379

**Published:** 2022-01-13

**Authors:** Paweł Rajewski, Dorota Zarębska-Michaluk, Ewa Janczewska, Andrzej Gietka, Włodzimierz Mazur, Magdalena Tudrujek-Zdunek, Krzysztof Tomasiewicz, Teresa Belica-Wdowik, Barbara Baka-Ćwierz, Dorota Dybowska, Waldemar Halota, Beata Lorenc, Marek Sitko, Aleksander Garlicki, Hanna Berak, Andrzej Horban, Iwona Orłowska, Krzysztof Simon, Łukasz Socha, Marta Wawrzynowicz-Syczewska, Jerzy Jaroszewicz, Zbigniew Deroń, Agnieszka Czauż-Andrzejuk, Jolanta Citko, Rafał Krygier, Anna Piekarska, Łukasz Laurans, Witold Dobracki, Jolanta Białkowska, Olga Tronina, Magdalena Wietlicka-Piszcz, Małgorzata Pawłowska, Robert Flisiak

**Affiliations:** 1 Department of Internal and Infectious Diseases, Provincial Infectious Disease Hospital, 85-030 Bydgoszcz, Poland; 2 Department of Infectious Diseases, Voivodship Hospital, Jan Kochanowski University, 25-369 Kielce, Poland; dorota1010@tlen.pl; 3 Hepatology Outpatient Clinic, ID Clinic, 41-400 Mysłowice, Poland; e.janczewska@poczta.fm; 4 Department of Internal Medicine and Hepatology, Central Clinical Hospital of the Ministry of Internal Affairs and Administration, 02-507 Warsaw, Poland; andrzejgietka@tlen.pl; 5 Clinical Department of Infectious Diseases, Specialist Hospital in Chorzów, Medical University of Silesia, 40-055 Katowice, Poland; wlodek.maz@gmail.com; 6 Department of Infectious Diseases and Hepatology, Medical University of Lublin, 20-059 Lublin, Poland; magdalena.tudrujek@gmail.com (M.T.-Z.); tomaskdr@poczta.fm (K.T.); 7 Regional Center for Diagnosis and Treatment of Viral Hepatitis and Hepatology, John Paul II Hospital, 31-202 Kraków, Poland; t.belica@szpitaljp2.krakow.pl (T.B.-W.); b.cwierz@szpitaljp2.krakow.pl (B.B.-Ć.); 8 Department of Infectious Diseases and Hepatology, Faculty of Medicine, Collegium Medicum Bydgoszcz, Nicolaus Copernicus University, 87-100 Toruń, Poland; d.dybowska@wsoz.pl (D.D.); w.halota@wsoz.pl (W.H.); mpawlowska@cm.umk.pl (M.P.); 9 Pomeranian Center of Infectious Diseases, Department of Infectious Diseases, Medical University of Gdańsk, 80-210 Gdańsk, Poland; lorbe@gumed.edu.pl; 10 Department of Infectious and Tropical Diseases, Jagiellonian University Collegium Medicum, 30-252 Kraków, Poland; sitkomar@o2.pl (M.S.); agarlicki@gmail.com (A.G.); 11 Hospital for Infectious Diseases in Warsaw, 01-201 Warsaw, Poland; hberak@wp.pl (H.B.); ahorban@zakazny.pl (A.H.); 12 Department of Infectious Diseases and Hepatology, Wrocław Medical University, 50-367 Wrocław, Poland; orlowska_iwona@yahoo.pl (I.O.); krzysimon@gmail.com (K.S.); 13 Department of Infectious Diseases, Hepatology and Liver Transplantation, Pomeranian Medical University, 70-204 Szczecin, Poland; theville@wp.pl (Ł.S.); martaws@wp.pl (M.W.-S.); asklepiada@wp.pl (Ł.L.); 14 Department of Infectious Diseases, Medical University of Silesia in Katowice, 41-902 Bytom, Poland; jerzyjr@gmail.com; 15 Ward of Infectious Diseases and Hepatology, Biegański Regional Specialist Hospital, 91-347 Łódź, Poland; deronzb@op.pl; 16 Department of Infectious Diseases and Hepatology, Medical University of Białystok, 15-089 Białystok, Poland; agnieszkaca@op.pl (A.C.-A.); robert.flisiak1@gmail.com (R.F.); 17 Medical Practice of Infections, Regional Hospital, 10-561 Olsztyn, Poland; citkoj@wss.olsztyn.pl; 18 Infectious Diseases and Hepatology Outpatient Clinic NZOZ “Gemini”, 62-571 Żychlin, Poland; rafalkrygier@gmail.com; 19 Department of Infectious Diseases and Hepatology, Medical University of Łódź, 90-419 Łódź, Poland; annapiekar@gmail.com; 20 Multidisciplinary Regional Hospital in Gorzów Wielkopolski, 66-400 Gorzów Wielkopolski, Poland; 21 MED-FIX Medical Center, 53-522 Wrocław, Poland; wdobracki@wp.pl; 22 Department of Infectious and Liver Diseases, Medical University of Łódź, 90-419 Łódź, Poland; jbialkowska@pro.onet.pl; 23 Department of Transplantation Medicine, Nephrology, and Internal Diseases, Medical University of Warsaw, 02-091 Warsaw, Poland; olgatronina@wp.pl; 24 Department of Theoretical Fundations of Biomedical Sciences and Medical Informatics, Nicolaus Copernicus University, 87-100 Toruń, Poland; mpiszcz@wp.pl

**Keywords:** hepatitis C virus, genotype HCV, diabetes

## Abstract

HCV infection is one of the main reasons for liver cirrhosis and hepatocellular carcinoma. In recent years, one finds more and more extrahepatic manifestations of HCV infection, including its possible influence on the development of diabetes. In the presented work, one finds the frequency analysis of the incidence of diabetes among 2898 HCV infected patients treated in Poland, and the assessment of their relevance to the HCV genotype and the progression of fibrosis. The results indicate that the hepatitis C infection seems to be a risk factor for diabetes in persons with more advanced liver fibrosis, for older people, and for the male gender. Thus, one found no differences regarding the frequency of its incidence depending on HCV genotype, including genotype 3.

## 1. Introduction

Chronic hepatitis C virus is one of the main reasons for chronic liver diseases globally and one of the most often occurring reasons for liver cirrhosis and hepatocellular carcinoma (HCC), contributing to a decrease in life quality and longevity.

It is commonly thought that there are six main HCV genotypes marked from 1 to 6. The prevalence varies worldwide. In Poland, one finds the dominating HCV genotype 1b in about 80%, whereas genotype 3 refers to 14%, and genotype 4 refers to 5% of infected patients.

The danger associated with the infection results from a huge virus spread in the population, long-term affliction with few symptoms or even an asymptomatic course of the disease, low detection rate, lack of vaccination, and neglect at health care units where infections occur most often (about 80% of cases).

In 80% the HCV infection transforms into the chronic form. About 20% of patients develop cirrhosis after 20 years of disease history, and 1–5% develop hepatocellular *carcinoma*.

Recent studies have proved that hepatitis C infection also contributes to the development of various metabolic disorders compared with healthy persons. A noticeable influence of HCV on the development of obesity, insulin resistance, carbohydrate disturbances, lipid disturbances, and hepatic steatosis resulted in the fact that metabolic disturbances in the course of the infection are named ‘metabolic and virus syndrome’ by some authors, whereas hepatic steatosis is described as an organic form of metabolic syndrome [1,2,3,4,5,6].

The introduction of HCV treatment with DAA-direct-acting antiviral therapy significantly improved the prognosis of HCV patients, reducing the rate of liver cirrhosis, HCC development, the number of liver transplants, and metabolic disorders in the course of the disease. Recent studies have shown that the type of DAA treatment is also important, especially for the development of HCC [7,8,9].

Diabetes is a chronic metabolic disease characterized by hyperglycemia resulting from disturbances in insulin secretion and effects.

It is estimated that there are 387 million people who have diabetes worldwide. About 3 million live in Poland. According to a report by the Assessment Panel of diabetes epidemiology in Poland from 2016, 2.7 million people suffer from diabetes, 1.22 million being women and 0.96 million being men, representing 6.1% of the female and 5.1% of the male population. Taking into account the estimated number of persons who suffer from the disease but are not diagnosed (36% of men and 15% of women), the total number of persons who have diabetes in Poland may be equal to 1.5 million men and 1.44 million women, representing 7.6% of the population of adult Poles. Autoimmune diabetes constitutes about 5%, type-II diabetes 85–90%, and the remaining portion constitutes other forms of diabetes (NATPOL, Receptometr Sequence; NHF report) [10,11,12,13,14,15,16].

## 2. The Aim of the Work

This study aimed to evaluate the influence of the HCV genotype on the prevalence of diabetes mellitus, to evaluate the overall incidence of diabetes in HCV patients, and to attempt to assess their relationship with the progression of liver fibrosis.

## 3. Material and Method

The studies covered 2898 patients with chronic HCV, including 1486 women (51%) and 1412 men (49%) with an age range of from 19 to 91 (median age 58), who were qualified for antiviral treatment based on the EpiTer multicentre study database (Table 1 and Table 2).

The analysis of medical documentation was carried out regarding physical examination according to the incidence and prevalence of diabetes. The HCV genotype was defined for each patient. Liver fibrosis was assessed by means of a liver biopsy and elastography. In the analyzed work, the definition of HCV genotype 1 includes genotype 1a and 1b together.

## 4. Results

Table 3 shows the characteristics of patients with diagnosed diabetes and patients who do not suffer from it. The analysis proves that the incidence of diabetes is associated with the old age of patients—*p* < 0.001, with male gender—*p* < 0.001, and with the progression of liver fibrosis—*p* < 0.001. Patients with more advanced liver fibrosis more often suffer from diabetes.

Further data analysis was carried out by means of logistic regression (Table 4 and Table 5) in order to determine independent factors related to the prevalence of diabetes.

The results of logistic regression proved that men more often suffered from diabetes than women—OR = 1.87 (1.5–2.32) as far as persons suffering from HCV are concerned, and more frequent incidence of diabetes was associated with the progression of fibrosis: OR = 2 (1.55–2.57) and older age: OR = 1.04 (1.03–1.05).

The obtained results have not proved the relevance between the prevalence of diabetes and the HCV genotype.

## 5. Discussion

The liver helps maintain a correct serum glucose level through the following processes one may find in hepatocytes: glycogenolysis, glycogenesis, glycolysis, and gluconeogenesis.

In case of low glucose value, or a deficiency of glucose in an organism, a liver synthesizes it using the processes of gluconeogenesis and glycogenolysis, whereas in the case of hyperglycemia or an excess of glucose in the food, the liver stores it in the form of glycogen, as well as in the organs and fat tissue in the form of triglycerides [10,17].

The liver is also a place of insulin degradation. The insulin level of liver blood flow through portal circulation is much higher than the level in system circulation, and is mostly subject to an uptake, even when first flowing through the liver, binding with insulin receptors on the surface of hepatocytes [10,18,19].

It has been proved that the development of insulin resistance and the disorders in the economy of carbohydrate associated with it depends on the degree of liver damage (hepatocytes) [19,20,21,22].

As far as the analysis is concerned, the prevalence of diabetes in patients suffering from chronic hepatitis C virus was found more frequently than in the general population group—16.7% vs. 7.6%. In the study group, 19.5% of men suffered from it and 14% of women. According to multicenter population studies (NATPOL, Receptometr, NHF report), 6.1% of women and 5.1% of men have diabetes (diagnosed by a doctor notwithstanding the treatment), whereas taking into account the additional estimate of patients with diabetes so far undiagnosed (about 36% of the men and 15% of the women), the total number of persons suffering from diabetes in Poland may constitute 7.6% of the adult Polish population with a higher incidence in men [12,13,14,15].

The increased interest of people with diabetes in the study group may provide proof of HCV’s influence on the development of carbohydrate disorders, including diabetes. Owing to the observations which prove that there is a greater number of men suffering from diabetes, one may expect that, taking into account undiagnosed and unexamined persons, diabetes is found more frequently in men than women.

Epidemiological data also point out the relevance of HCV infection and the development of carbohydrate economy disorders—impaired fasting glucose, glucose intolerance, or even diabetes. As estimated, the prevalence frequency of carbohydrate disorders in patients with chronic hepatitis C virus is 4 to 10 times higher than in the population of healthy persons, and occurs in 14% to 30% of patients. As for the population study (Third National Health and Nutrition Examination Survey [NHA-NES III]), patients aged 40 and more with HCV suffered 3 times more frequently from diabetes than healthy people did, whereas the prevalence of diabetes in patients with diabetes risk factors could be even 11 times higher, as in the analyzed group of patients [23,24,25]. Another case study conducted by Custro and partners proved the frequency of diabetes to be 4 times higher in patients with HCV compared to the population of healthy persons. In the study group, 40% of persons had impaired glucose tolerance (11.7% in the general population), and 7% were suffering from diabetes (4.9% in the general population) [18,25].

As far as the analyzed group was concerned, the incidence of diabetes was observed more frequently in older people—OR = 1,04 (1.03–1.05). More frequent diabetes prevalence is also associated with the patient’s age, especially persons >65, where it occurs 25–30%. According to POLSENIOR studies, 18% of patients aged 65 and over suffered from diabetes, whereas impaired fasting glucose level and glucose intolerance were found in 20% [12,14].

Insulin resistance in old age is associated with impaired compensation mechanisms related to decreased insulin secretion by the pancreas. Moreover, together with age progression, the total mass of fat tissue increases, muscle tissue decreases. One also observes the increase of abdominal obesity, change of eating habits, a decrease in physical activity, as well as the possible influence of medicines and underlying diseases, which may affect the development of diabetes.

As far as the study is concerned, one finds a more frequent incidence of diabetes in patients with the chronic hepatitis C virus together with the progression of cirrhosis of the liver—OR = 2(1.55–2.57). The impairment of liver function in 50% of cases, and in the case of liver cirrhosis in 80%, contributes to the development of glucose intolerance, and in 10% to the development of diabetes. The patients suffer from carbohydrate disorders even when experiencing a higher serum insulin level which may show tissue resistance to insulin secretion—insulin resistance. The development of insulin resistance in liver diseases results from the defect of liver parenchyma and the decrease of glucose metabolism capacity. The defect of glucose metabolism of patients with liver cirrhosis results also from receptor hepatocytes defects. The defect of active parenchyma also compromises the metabolism of hormones that increase blood glucose levels, those which are insulin antagonists such as glucagon and growth hormone [17,26].

Based on long-term observations, it has been proved that the following risk factors result in the development of diabetes in patients with HCV: old age, HCV genotype 3, serious liver fibrosis or cirrhosis, positive diabetes family history, and kidney or liver transplants [17,26,27].

In our study, no relevance was found between HCV genotype and the incidence of diabetes in the study group of patients with chronic hepatitis C virus. This may result from the low patient rate, patients with the HCV genotype of other than 1 taking part in the study, and from the population genotype distribution occurring in Poland.

Low interest of patients with the HCV genotype 3 (7.5%) may contribute to it as well, a genotype especially associated with the occurrence of metabolic disorders-insulin resistance, diabetes, lipid disorders, arteriosclerosis, and hepatic steatosis.

The increase of the diabetes risk factor as far as hepatitis C virus is concerned seems to be associated with insulin resistance and chronic inflammatory reaction resulting from the increased synthesis of pro-inflammatory cytokines—mainly Tumour Necrosis Factor (TNF) alfa and interleukin 6 (IL-6). The increase of insulin level results from insulin resistance, insulin which is not effectively used by tissues, secondary hyperinsulinemia, and, consequently, the increase of the serum glucose level and the development of carbohydrate disorders. Insulin resistance also increases the lipolysis of fat tissue and the access of free fatty acids, leading to live steatosis. The process of insulin resistance refers mainly to all HCV genotypes, however, in the case of HCV genotype 3, it is expressed the most and co-exists with a lower rate of HOMA index [17,26].

The development of insulin resistance is also associated with the direct virus influence on the insulin signaling pathway. As far as HCV genotype 3 is concerned, the lipids of the virus may directly affect intra-hepatic insulin signaling through the expression decrease of a peroxisome proliferator-activated receptor (PPAR alfa). It controls the expression of genes of mitochondrial palmitoyltransferase-1 carnitine (CPT-1), which reduces mitochondrial beta-oxidation responsible for the catabolism of fatty acids and acetyl oxidase CoA (AOX).

One of insulin’s resistance mechanisms in patients with chronic liver diseases is acquired resistance to growth hormone (GH), which results from the increase of pro-inflammatory cytokines, mainly of TNF-alfa [28,29,30,31].

Acquired resistance to GH results, in consequence, in the decrease of insulin-like growth factor level 1 (IGF-1) and GH compensatory growth, which secondarily increases insulin resistance.

Insulin resistance through the increased flow of free fatty acids (FFA) to a liver, hypertriglyceridemia, and hyperinsulinemia contributes to severe hepatic steatosis and cirrhosis. In recent studies, it has been proved that the reduction of insulin receptor substrate 1 (IRS-1) and Kinase B (PKB/Akt) plays a vital role regarding the origin pathomechanism of insulin resistance in patients with HCV is concerned. Moreover, HCV affects the SOCS-3 suppressor of cytokines signaling proteins which are directly stimulated by HCV core proteins, and contribute to the degradation of IRS-1 and IRS-2 by the conduction inhibition in the insulin signaling pathway [32].

Core proteins in HCV genotype 3 affect IRS-1 degradation through the decrease of synthesis of peroxisome proliferator-activated receptor-gamma (PPAR gamma) and the stimulation of the synthesis of SOCS-7 regulatory protein. Insulin resistance is also associated with the increased level of pro-inflammatory cytokines—TNF alfa, IL-6. The presence of insulin resistance in the metabolic liver steatosis is co-dependent on its increase and does not prove to be a beneficial factor determining fibrosis progression [33,34,35,36,37,38].

The risk increase of diabetes development is also associated with the increased release of a leptin, resistance, and decreased release of adiponectin by fat tissue [39,40,41].

Patients with HCV may also have obesity, metabolic syndrome, or NAFLD, which contributes to insulin resistance and the development of diabetes. The mechanisms of insulin resistance and diabetes are complex in patients with liver disease, but also depend on their etiology. Complex mechanisms are found primarily in patients with NAFLD or obesity, but NAFLD or obesity may overlap with HCV infection, and then we potentially have several pathophysiological mechanisms of disorders in glucose metabolism-primary metabolic and secondary “viral”. Diabetes mellitus type 2 or obesity also leads to the development of fatty liver disease, steatohepatitis, or cirrhosis due to fatty liver disease in HCV patients. Additionally, diabetes or obesity are factors leading to faster progression of fibrosis [42].

The common mechanism of prediabetes and diabetes in patients with obesity, metabolic syndrome, NAFLD, or HCV is insulin resistance. Insulin resistance in obesity, metabolic syndrome, and NAFLD is closely associated with increased visceral adipose tissue mass. This latter shares a directly proportional release of mediators from adipocytes-leptin and resistin, which inhibit the sensitivity to insulin action. Insulin resistance determines an accumulation of free fatty acids (FFAs) inside the liver, due to increased hepatic lipogenesis and the missed suppression of lipolysis of the adipose tissue, and the accumulation of intrahepatic fat. Determining, in turn, a modification of insulin signaling pathways, thus worsening the systemic state of insulin resistance. Genetic and epigenetic factors, diet, lifestyle, chronic inflammation, oxidative stress, and microbiota may also be involved in the development of prediabetes, diabetes, and NAFLD [42].

Diabetes and obesity are also independent factors of HCC development, especially in HCV patients. Obesity, NAFLD, diabetes mellitus type 2, or the metabolic syndrome overlap on HCV infections also contribute to a faster fibrosis progression and may cause a worse response to HCV treatment [42,43]. The liver biopsy is very important in the differential diagnosis, especially for the determination of NASH and cirrhosis of the liver due to metabolic steatosis-insulin resistance, type 2 diabetes, or metabolic syndrome in differentiation with changes in the course of HCV [44].

The incidence of diabetes is also associated with a poor response to anti-virus treatment. Whereas effective treatment results in the decrease of insulin resistance and decreased level of fasting blood glucose, which may suggest a direct influence of HCV on its development [7,45,46,47]. Metabolic disorders in HCV patients, especially insulin resistance, may cause a poorer response to DAA therapy due to the formation of RAS-Resistance associate mutations [48].

Recent studies—2426 patients with HCV, 42% of whom had liver fibrosis stage F0-F2 and 58% of whom had liver fibrosis stage F3–F4—have shown that effective HCV treatment with DAA was associated with significant reductions in HOMA-insulin resistance and HOMA-β-cell function, an increase in HOMA-insulin sensitivity, and a reduction of the prevalence of diabetes in this group of patients [9].

Effective treatment of HCV with DAA also reduces the incidence of cardiovascular disease and events [49]. A study on 770 HCV-positive prediabetic patients showed that treatment with DAA reduces the risk of cardiovascular events [50].

Chronic hepatitis C virus is still a serious problem for contemporary medicine. On the one hand, detection comes too late due to the asymptomatic course of the disease. On the other hand, there is the risk of developing serious complications associated with the progression of the infection. There are also some extrahepatic symptoms of HCV infection, which contribute to, among others, metabolic disorders observed throughout the course of the disease.

The development of liver steatosis, obesity, insulin resistance, and accompanying diabetes, as well as lipid disorders, contribute to the progression of fibrosis, faster development of liver cirrhosis, and hepatocellular carcinoma; as well as worsening the response to anti-virus treatment, which constitutes some reinforcement and protection element of a virus.

As far as chronic hepatitis C virus is concerned, one finds some characteristic elements for a so-called metabolic syndrome, that is, a set of risk factors of cardiovascular diseases, owing to the fact that HCV infection has been treated in the last years as a metabolic liver disease which may also be a risk factor for arteriosclerosis and cardiovascular diseases; coronary artery disease, a stroke, and peripheral vascular disease.

Presently, it is still being discussed whether HCV is a risk factor of diabetes, and whether diabetes is a risk factor of extrahepatic manifestation of infection.

In the light of current research, it should be concluded that HCV infection is a risk factor for developing insulin resistance and diabetes, regardless of the HCV genotype, and that effective DAA treatment reduces their incidence. Therefore, screening for HCV infection in diabetic patients is important, as well as active screening for HCV infections in the general population for early detection and treatment, which may reduce the incidence of diabetes.

## 6. Conclusions

1. Hepatitis C infection is a risk factor for insulin resistance and diabetes.
2. The age of the patient, male gender, and the progression of fibrosis are associated with more frequent incidences of diabetes in patients suffering from HCV.
3. The HCV genotype does not affect the prevalence frequency of diabetes, and HCV genotype 3 infection does not affect the risk increase of diabetes.
4. The elimination of HCV may contribute to reducing the incidence of diabetes, too.

## Figures and Tables

**Table 1:** The characteristics of the study group.

Total Number		*n* = 2898
		*n* (%)
**Gender**		
	female	1486 (51.28)
	male	1412 (48.72)
**Genotype**		
	1	2596 (89.58)
	3	219 (7.56)
	4	83 (2.86)
Fibrosis (F) **		
	1	624 (22.74)
	2	391 (14.25)
	3	462 (16.84)
	4	1267 (46.17)
**Age (years)**		58 (19–91) *

* Median (Range). ** Liver fibrosis was not determined for 154 persons.

**Table 2:** Age distribution depending on HCV fibrosis.

Age	Fibrosis 0–2 n-1015	Fibrosis 3–4 n-1729
	Median (Range)	Median (Range)
	52 (19–84)	59 (21–91)

**Table 3:** The characteristics of patients and the incidence of diabetes.

		Overall	Diabetes Yes *n* = 483	Diabetes No *n* = 2415	
		*n* (%)	*n* (%)	*n* (%)	*p*
gender					
	female	1486 (51.28)	208 (14)	1278 (86)	
	male	1412 (48.72)	275 (19.5)	1137 (80.5)	<0.001
fibrosis					
	1	624 (22.74)	52 (8.3)	572 (91.7)	
	2	391 (14.25)	40 (10.2)	351 (89.8)	
	3	462 (16.84)	73 (15.8)	389 (84.2)	
	4	1267 (46.17)	278 (21.9)	989 (78.1)	<0.001
fibrosis					
	0–2	1015 (36.99)	92 (9.1)	923 (90.9)	
	3–4	1729 (63.01)	351 (20.3)	1378 (79.7)	<0.001
genotype					
	1	2596 (89.58)	435 (16.8)	2161 (83.2)	
	3	219 (7.56)	41 (18.7)	178 (81.3)	
	4	83 (2.86)	7 (8.4)	76 (91.6)	0.094
age [years]		58 (19–91) *	62 (30–86) *	57 (19–91) *	<0.001

* Median (Range).

**Table 4:** Factors associated with the prevalence of diabetes—logistic regression. OR (CI): odds ratio with 95% confidence interval; SE: standard error of the estimate.

	Simple LR				Multiple LR			
	Estimate	SE	OR(CI)	*p*	Estimate	SE	OR(CI)	*p*
Gender M vs. F	0.3961	0.1005	1.49 (1.22–1.81)	0.0001	0.6248	0.1117	1.87 (1.5–2.32)	<0.0001
Age [years]	0.0408	0.0043	1.04 (1.03–1.05)	<0.0001	0.0433	0.0049	1.04 (1.03–1.05)	<0.0001
Fibrosis 3–4 vs. 0–2	0.9382	0.1246	2.56 (2–3.26)	<0.0001	0.6923	0.1284	2 (1.55–2.57)	<0.0001
Genotype 1 vs. 3	−0.1348	0.181	0.87 (0.61–1.25)	0.4566	−0.1496	0.1921	0.86 (0.59–1.25)	0.4361
Genotype 4 vs 3	−0.9166	0.4312	0.4 (0.17–0.93)	0.0335	−0.8808	0.4701	0.41 (0.16–1.04)	0.061

**Table 5:** Factors related to the incidence of diabetes—results of logistic regression OR (CI): odds ratio with 95% confidence interval.

	Simple LR		Multiple LR	
	OR(CI)	*p*	OR(CI)	*p*
Sex M vs. F	1.49 (1.22–1.81)	0.0001	1.87 (1.5–2.32)	<0.0001
Age [years]	1.04 (1.03–1.05)	<0.0001	1.04 (1.03–1.05)	<0.0001
Fibrosis 3–4 vs. 0–2	2.56 (2–3.26)	<0.0001	2 (1.55–2.57)	<0.0001
Genotype 1 vs. 3	0.87 (0.61–1.25)	0.4566	0.86 (0.59–1.25)	0.4361
Genotype 4 vs. 3	0.4 (0.17–0.93)	0.0335	0.41 (0.16–1.04)	0.061

## Data Availability

Data supporting reported results can be provided upon request from the corresponding author.
